# Circulating tumor DNA: current challenges for clinical utility

**DOI:** 10.1172/JCI154941

**Published:** 2022-06-15

**Authors:** Donna K. Dang, Ben H. Park

**Affiliations:** Vanderbilt-Ingram Cancer Center, Division of Hematology/Oncology, Department of Medicine, Vanderbilt University Medical Center, Nashville, Tennessee, USA.

## Abstract

Cancer cells shed naked DNA molecules into the circulation. This circulating tumor DNA (ctDNA) has become the predominant analyte for liquid biopsies to understand the mutational landscape of cancer. Coupled with next-generation sequencing, ctDNA can serve as an alternative substrate to tumor tissues for mutation detection and companion diagnostic purposes. In fact, recent advances in precision medicine have rapidly enabled the use of ctDNA to guide treatment decisions for predicting response and resistance to targeted therapies and immunotherapies. An advantage of using ctDNA over conventional tissue biopsies is the relatively noninvasive approach of obtaining peripheral blood, allowing for simple repeated and serial assessments. Most current clinical practice using ctDNA has endeavored to identify druggable and resistance mutations for guiding systemic therapy decisions, albeit mostly in metastatic disease. However, newer research is evaluating potential for ctDNA as a marker of minimal residual disease in the curative setting and as a useful screening tool to detect cancer in the general population. Here we review the history of ctDNA and liquid biopsies, technologies to detect ctDNA, and some of the current challenges and limitations in using ctDNA as a marker of minimal residual disease and as a general blood-based cancer screening tool. We also discuss the need to develop rigorous clinical studies to prove the clinical utility of ctDNA for future applications in oncology.

## Introduction

**Figure 1:**
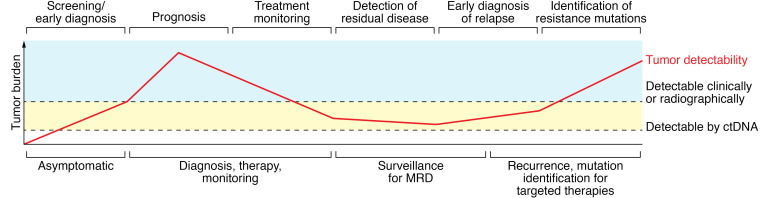
The potential utility of ctDNA across the spectrum of human cancers. MRD, minimum residual disease. Adapted with permission from *Cancers* (108).

Liquid biopsies have emerged as a new method for analyzing biomarkers from blood and other bodily fluids. Today, “liquid biopsy” usually refers to the identification and analysis of cell-free DNA (cfDNA) derived from patient plasma samples. cfDNAs are free-floating, naked DNA molecules that are shed into the circulation actively by cells, as well as by cells undergoing death by apoptosis and/or necrosis (1). Many studies have shown the potential benefit of using cfDNA for clinical management in such areas as identification of fetal genetic anomalies (2), solid organ transplant rejection (3), and identification of cancer mutations from blood (4). For cancer and oncology research, most studies evaluating cfDNA have examined DNA derived specifically from cancer cells, also known as circulating tumor DNA (ctDNA). In this relatively brief Review, it is not feasible to discuss all existing and emerging applications of cfDNA in helping guide clinical decision making. Numerous excellent recent reviews have highlighted the many clinical studies using ctDNA as integral and integrated biomarkers (5–7). Thus, we will focus on the technical aspects of ctDNA, including the challenges of using ctDNA in oncology across the spectrum from primary prevention to metastatic disease (Figure 1).

The field of oncology has evolved at a rapid pace over the past twenty years. Many cancers that were once uniformly fatal can now be treated effectively for years, and some appear curable even in the metastatic setting (8–10). Unquestionably the discoveries of genetic alterations that lead to human cancers have allowed targeting of these alterations for therapeutic gain. Historically, sequencing of a patient’s tumor to find mutations was laborious, time-consuming, and expensive and could only be performed on a limited scale. Newer technologies have largely overcome these barriers but have also led to new challenges. In this Review, we first describe the rationale of identifying genetic alterations in cancers that are amenable to targeted and other therapies, including a brief background on the principles of oncology and the goals and differences in treating solid-tumor patients with early-stage versus metastatic disease. We then briefly review the history of liquid biopsies, cfDNA, and ctDNA, and the various technologies and platforms for analyzing these substrates. We also include limited examples of past and current studies that have led to routine use of ctDNA in the management of patients with cancer. Finally, we present some of the challenges of achieving clinical utility of liquid biopsies/ctDNA to address pressing unmet needs in oncology.

## Precision oncology in early-stage and metastatic cancer

Precision oncology arose from the idea that understanding the molecular underpinnings of a patient’s tumor creates an opportunity to use drugs to selectively target cancer cells harboring these genetic changes (11). These genetic alterations, which are generally gene mutations, amplifications, fusions, and loss of tumor suppressor genes, are termed “drivers,” since they are responsible for cancerous phenotypes including the ability to proliferate and metastasize (12, 13). However, drivers also represent the Achilles’ heel of cancers, as the majority of these genetic alterations are somatic, i.e., present only in the cancer cell. This affords the opportunity to develop therapies with exquisite specificity for cancer cells while leaving normal cells relatively unharmed. Although there are indeed heritable mutations/genetic alterations that predispose to cancer and are therefore found in every cell that contains DNA (e.g., germline mutations in genes such as *BRCA1* and *BRCA2*), these represent a minority of all cancer cases (14). Notably, even in *BRCA1* and *BRCA2* gene mutation carriers, subsequent somatic genetic events must occur to transform a normal cell into a cancer cell (15) that can also result in therapeutic vulnerabilities. An example of this is PARP inhibitors, which have been approved for breast cancers in both the metastatic and the early-stage setting for patients with germline pathogenic *BRCA1* or *BRCA2* mutations (16–20), and for ovarian, pancreatic, and prostate cancers with *BRCA1* or *BRCA2* mutations (21–26).

The ability to assess molecular alterations in tumor tissues was limited in the past because of the cost and time of “first-generation sequencing.” With the advent of next-generation sequencing (NGS), rapid analysis of a cancer genome became feasible (27). Currently, however, most clinical cancer gene NGS assays consist of panels of 500 to 600 genes, though whole-exome and whole-genome NGS is possible. The reasons for this are pragmatic: currently there is little if any clinical actionability in obtaining more than 500 to 600 genes of a patient’s tumor via NGS, and performing whole-exome/whole-genome NGS increases costs and, importantly, turnaround time owing to the additional bioinformatics analyses required. That said, NGS of tumors along with the use of targeted therapies and immunotherapies has become standard of care for most if not all cancers in the metastatic setting and is also now indicated for select early-stage cancers such as non–small cell lung cancer and melanomas (28, 29). This topic is covered in more depth in other Reviews in this series (30), and therefore we will only touch upon this topic as needed for clarity and background.

To demonstrate how liquid biopsies are currently being used, and how their future use could address unmet needs, we will next review the goals and principles of systemic therapies as they relate to patients with solid tumors (31). For the majority of solid tumors, stage IV represents metastatic disease and is considered incurable in most cases. Therefore therapies are directed toward treating the cancer to extend and improve quality of life. Currently this is where the bulk of precision oncology efforts are focused: to find molecular alterations that have targeted therapies (11, 32, 33). Also, because metastatic disease is largely incurable, there tends to be more latitude in pursuing therapies that may not be standard of care but may realistically still afford a chance of benefit (11, 31, 32, 34). Such strategies, however, must be considered carefully and are nowadays often discussed at molecular tumor boards (33), and have led to guidelines to contextualize a framework for prioritizing actionable NGS results (35).

For patients with early-stage cancer, the role of systemic therapies like chemotherapy is additional treatment after local therapy (surgery and/or radiation) in patients who would otherwise later relapse with incurable metastatic disease due to the presence of microscopic or minimal residual disease (MRD) (36). Although clinical trials clearly provide proof that these additional, also known as adjuvant, systemic therapies improve cure rates for early-stage cancers, it is also well known and accepted that significant populations of patients who are already cured after local therapies receive systemic therapies needlessly, thus being exposed to their potential toxicities. This, in fact, led to the development of a genomic test for breast cancer to help identify such patients (37). Moreover, considerable numbers of patients will still develop incurable metastatic disease at some future point despite having received adjuvant systemic therapies. Equally important, owing to the curative intent of therapy for early-stage disease (38), adding, removing, or replacing adjuvant therapies outside of a clinical trial is generally discouraged, as there is great concern that doing so could compromise the chance of cure. Thus, adding targeted therapies for cancers with certain mutations in early-stage disease will generally require a rigorous level of evidence from large prospective randomized controlled trials with multi-year follow-up, as was recently shown for PARP inhibitors and *BRCA1*- or *BRCA2*-derived breast cancers (18, 20). Hence a true unmet need for precision oncology and ctDNA use in early-stage cancers is determining who is cured, who is not, and whether additional therapies beyond standard of care could cure additional patients who still have MRD.

## Liquid biopsies and ctDNA

The advent of liquid biopsies changed the manner in which oncologists approach cancer therapies. There are now many genomic and genetic alterations that influence treatment decisions, and liquid biopsies, specifically ctDNA, allow for a relatively easy, noninvasive way to identify these alterations. We acknowledge that “liquid biopsy” can also refer to other analytes in blood used to assess cancer burden and/or genomic and genetic features of a patient’s cancer, including circulating tumor cells (39), protein biomarkers (40), and cell-free RNA (cfRNA) (41). Development of new DNA detection technologies for ctDNA, as well as the ever-increasing number of targeted therapies and immunotherapies being approved for cancers, has allowed for a more refined approach to treating patients with various malignancies. Although this approach has been mostly used in patients with metastatic disease for the reasons outlined above, recent studies support the use of targeted therapies in the adjuvant setting (18, 29).

Though NGS of tumor tissue is generally preferred, often a tissue biopsy is unobtainable or of insufficient quantity or quality, and therefore ctDNA analysis using blood is the only available option. Additionally, there are clinical situations in which procurement of tumor tissue for NGS is delayed because of the time needed to set up a biopsy or surgery, as well as time for processing of tissues for pathologic assessment. These factors further add to the turnaround time for obtaining NGS results from tumors (42). In contrast, liquid biopsies generally require only phlebotomy using several blood tubes, and samples can be quickly sent off for NGS analysis. In addition, liquid biopsies allow for a fast and easy method of serial analysis over multiple time points that can help guide treatment decisions by identifying the emergence of mutations and other genetic alterations that predict for resistance or response to the next line of therapy (43, 44). Moreover, this approach can avoid targeted therapies and immunotherapies that would have little chance of benefit, thereby minimizing toxicities, financial burden, and other adverse events.

Although the ability to use cfDNA for clinical applications is relatively new, the existence of cfDNA was first described in 1948 (45). cfDNA refers to naked free-floating DNA molecules shed or released from cells, which occurs under normal circumstances in healthy tissues due to cell turnover and active shedding into the circulation (46). Presently, there is no clear known function of cfDNA, and many feel it may simply be a cellular waste product that eventually clears the circulation through the kidneys as urine cfDNA. In addition, the half-life of cfDNA is short, speculated to be 1 to 2 hours (47), maybe because of DNases in the blood that lead to quick degradation. cfDNA is typically found as short fragments approximately 160–180 base pairs in length and is now used for a number of clinical applications, such as assessing fetal genetic anomalies from maternal blood (48). Interestingly, cfDNA was initially proposed as a way to assess cancer patients’ overall tumor burden, as it was noted that, in general, higher overall cfDNA levels are detected in patients with cancer than in individuals without cancer (49). Ultimately, total cfDNA levels were not sensitive nor specific enough for following a patient’s overall tumor burden and response to therapies.

Regardless, ctDNA assessment has become relatively routine in patients with metastatic disease, and ctDNA generally can be present among cfDNA at variant allele fractions (VAFs) ranging from <0.1% to 10%, though it can be even higher (42, 50). VAFs represent the percentage of mutant DNA molecules relative to the total number of DNA molecules for a given gene. Numerous studies have demonstrated that higher VAFs are associated with increased tumor burden and worse prognosis (51–54). Importantly, therapeutic intervention can also lead to changes in the levels of ctDNA in the blood (47). For example, surgical resection of tumors can lead to a decrease in ctDNA, as can chemotherapy, targeted therapy, and immune therapies (42, 47, 55, 56). Collectively, these and now numerous clinical studies have demonstrated that changes in ctDNA levels correlate with therapies, both local and systemic. There have also been numerous correlative studies evaluating whether de novo or acquired mutations found in ctDNA can predict response or resistance to a given therapy (57, 58) and/or whether serial ctDNA monitoring can detect MRD (43, 59, 60). The majority of these studies retrospectively evaluated samples that had been collected prospectively. However, due to the rather rigorous requirements for optimal analyte preparation (61), described below, as well as the need for sufficient plasma DNA for analysis, there are caveats to these studies, as only a few were prospectively planned to incorporate ctDNA analyses. More recently, studies have demonstrated some limited clinical validation for MRD detection using newer approaches (59, 62) as well as using ctDNA as a primary blood-based cancer screening test (40, 63). These studies also highlighted some of the limitations of ctDNA, including that some cancer types may secrete or shed less DNA into the circulation for unknown reasons (64).

## ctDNA collection and processing

Blood, and specifically plasma, is the ideal analyte for ctDNA collection and is currently being used in the clinic predominantly for companion diagnostic purposes, meaning finding mutations that are linked to an FDA-approved therapy. Although plasma is now the preferred analyte for cfDNA analyses, cfDNA can be detected in serum, though serum cfDNA has decreased integrity compared with plasma cfDNA (65–67). However, other sources of cfDNA/ctDNA have been evaluated, including cerebrospinal fluid (68) and urine ctDNA (55). For most current commercial and research assays, cfDNA is generally isolated by separation of plasma from whole blood using double centrifugation protocols to maximally remove cells, followed by extraction of nucleic acids using various techniques (69). It should be noted that great care must be taken in processing cfDNA and that the use of dedicated equipment (centrifuges, pipettes, etc.), “clean rooms,” dedicated hoods, etc. is essential to avoid contamination of samples with aerosolized DNA, which is a major issue in dealing with NGS and other assays that measure mutations at 0.01% VAF or lower (70). Generally speaking, at least 20 mL of whole blood should be obtained to ensure enough plasma DNA for analysis, though this will vary depending on the nature of the assay (71). Ideally, cfDNA should be isolated within hours of blood collection to prevent lysis of white blood cells; otherwise cell lysis will liberate large amounts of cellular genomic DNA into the plasma component within the blood collection tube (61, 72, 73). Because of the wide variation in cfDNA concentration between individuals, the amount of cfDNA needed to truly reach the technical limit of detection (LOD) of any given platform can be problematic. For example, if the technical LOD for a given assay is one mutant DNA molecule in 10,000 wild-type DNA molecules, then to truly define a negative result, the sample must contain at least 10,000 DNA molecules and preferably severalfold above this amount whenever possible. In practice, obtaining 10,000 DNA molecules or “genome equivalents” is non-trivial. A genome equivalent (GE) is defined as one genome of an organism, which in the case of humans is the haploid genome. The haploid genome is approximately 3 billion base pairs, and has a molecular mass of approximately 3 picograms. Therefore, to obtain 10,000 GEs in a given sample, there must be approximately 30 nanograms of DNA, which is often challenging given the relatively low concentrations of plasma DNA found in human samples (74).

As mentioned above, analyte considerations must be taken into account in the processing of plasma DNA from blood, including contamination issues and lysis of white blood cells when blood samples are not processed within a few hours of collection. We and others have shown that Streck Cell-Free DNA BCT tubes can prevent cellular lysis, owing to cell membrane–stabilizing preservatives used in these tubes (61, 74). Indeed, standard EDTA tubes that are not processed within hours can lead to inaccurate quantification of mutational allele frequencies due to lysis of blood cells, thereby increasing the number of wild-type alleles. Therefore, proper handling and timely sample processing are key steps to ensure the integrity of cfDNA for downstream analysis.

## Analysis of ctDNA

Methods to analyze ctDNA have historically used either PCR-based assays or NGS paired with specialized bioinformatics analysis. Although many PCR-based strategies and technologies have been created and are still available for cfDNA testing, a discussion is beyond the scope of this Review. Rather, we will briefly touch on the initial single-molecule PCR methods for analyzing cfDNA and how they evolved into the current use of NGS techniques.

Digital PCR was the initial method described by Kinzler and Vogelstein, which established the idea of single-molecule amplification that could allow assessment of rare mutant alleles in a background of many wild-type alleles (75). Although the term “digital” is often assumed to have an electronic connotation, it refers (to the binary nature of diluting samples such that each partition or compartment of the assay platform has either one or zero DNA molecules. The first high-throughput digital PCR method was called BEAMing (beads, emulsions, amplification, and magnetics; ref. 76). This somewhat cumbersome process was successfully employed to demonstrate that digital PCR could be used for ctDNA detection in metastatic disease and following response to therapies (4, 77). Subsequent commercial evolution of BEAMing came from droplet digital PCR (ddPCR), which enabled more uniform partitioning of DNA molecules in a technology platform that could be used by standard research laboratories (60). Many studies demonstrating the analytic and clinical validity of ddPCR followed (78–80). However, a major limitation of all digital PCR platforms is the finite number of mutations per assay that can be analyzed. But an advantage of digital PCR over current NGS methods is that the readout for digital PCR does not require any bioinformatics analyses and is akin to data collected and analyzed using a flow cytometer. This readout can significantly reduce turnaround time, which can be clinically useful. Moreover, the accuracy of digital PCR and the ability to easily run technical replicates allowed for an LOD of 0.01% to 0.001%, which at the time of digital PCR’s emergence was not possible with NGS. However, with the advent of “barcoding” of individual DNA molecules using molecular tags, along with new bioinformatics analyses, NGS of cfDNA evolved to overcome the limitations of errors introduced by PCR amplification and/or NGS, to allow for a technical LOD that equaled or even surpassed that of digital PCR (81).

NGS offers deeper and more comprehensive profiling methods to identify genetic alterations. Rather than querying for only known variants with molecular probes as with digital PCR, NGS has the capacity to find genetic perturbations in a relatively unbiased fashion, since sequencing, by its very nature, will identify all the base pairs of a given DNA molecule. The flexibility in selectively capturing and/or amplifying only regions of interest has led to approaches for sequencing only parts of a genome, including so-called “exome capture” and also gene panels (82). Unfortunately, NGS is prone to sequencing errors, due to the inherent nature of strand synthesis, as well as PCR amplification. In the past, this had hindered the use of NGS for ctDNA analysis, as allele fractions of ctDNA are often low, in the range of 0.01% to 1% ctDNA to cfDNA (42, 83).

**Figure 2:**
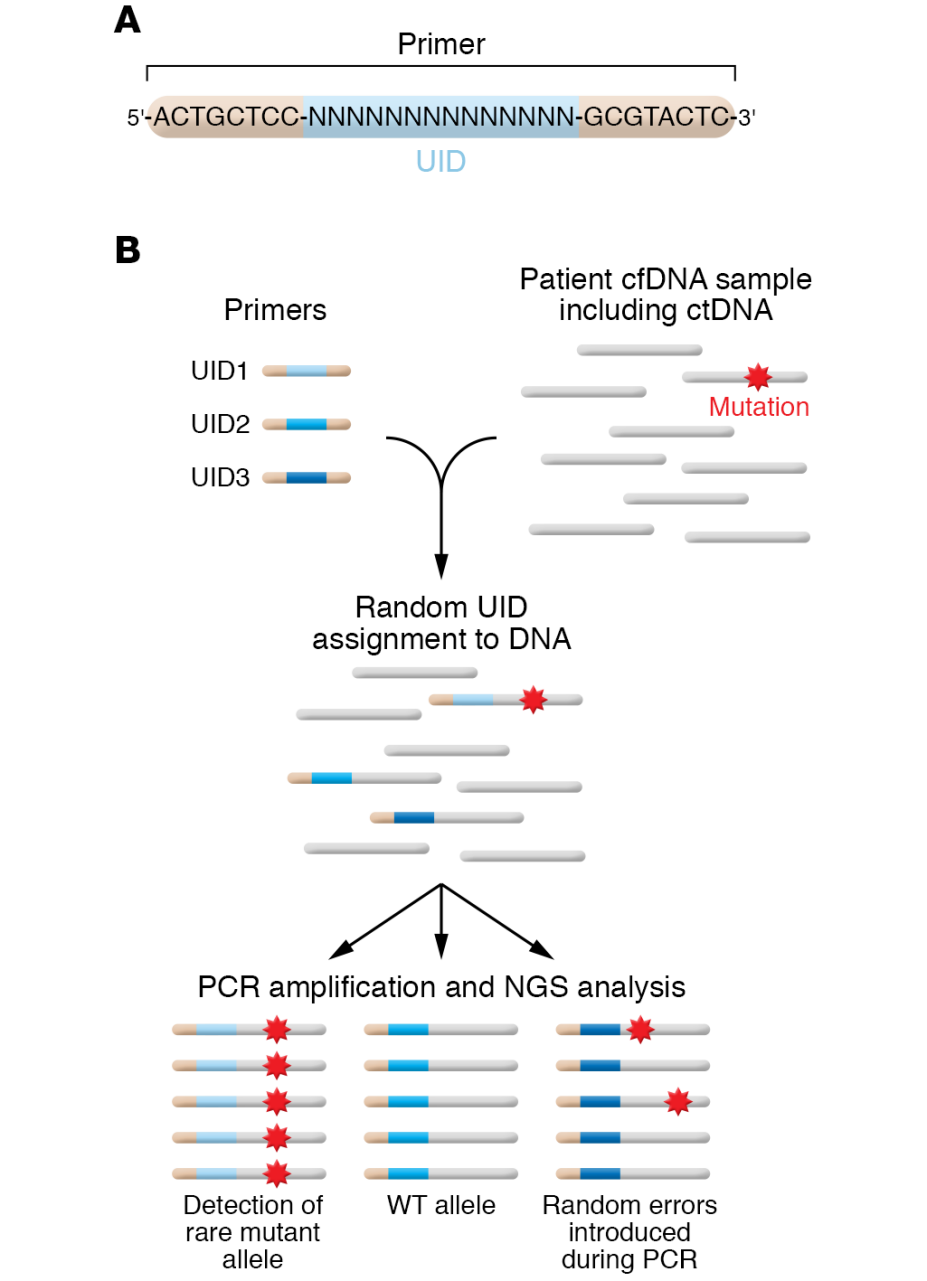
Molecular barcoding for NGS libraries to improve detection of rare mutations. (**A**) Incorporation of random sequences for degenerate primers used to molecularly tag each DNA molecule. Each “N” can be either A, C, G, or T and is chosen randomly during synthesis. (**B**) Schema of Safe-SeqS (adapted with permission from *Proceedings of the National Academy of Sciences of the USA*; ref. 84). Clinically relevant mutations are present at very low frequency in patient samples. Barcoding of DNA can improve the signal-to-noise ratio in NGS analysis, because mutant tumor alleles containing the same UID will be amplified, whereas random errors resulting from PCR amplification will remain at low frequency.

A seminal breakthrough in NGS was the concept of “tagging” each individual DNA molecule with a molecular barcode. The advent of barcoding of each individual DNA fragment with unique identifiers (UIDs), coupled with bioinformatics analysis and higher-fidelity DNA polymerases, allowed for ultrasensitive detection of ctDNA (84). The first such technique was the Safe-Sequencing System (Safe-SeqS; ref. 84), followed by many other variations, including cancer personalized profiling by deep sequencing (CAPP-Seq; ref. 85), tagged-amplicon deep sequencing (TAm-Seq; ref. 86), and Duplex Sequencing (Duplex-Seq; ref. 87). All of these incorporate some form of barcoding of individual DNA molecules that enables ultrasensitive detection of mutations that otherwise would be either below the LOD and/or impossible to distinguish from NGS/PCR artifacts. The basic concept is shown in Figure 2A. The simplest method of generating a UID is to design primers containing degenerate, random sequences. As an example, one could design 30-mer primers with fixed 5′ and 3′ ends, each with 8 nucleotides, but the remaining 14 internal nucleotides would be designated “N” and randomly picked to be either A, C, G, or T during synthesis (Figure 2B). Thus each base pair position has a 1 in 4 chance of being a given nucleotide. When multiplied by the 14 internal nucleotides (1/4 to the 14th power), this yields a combination of 268,435,456 unique primer sequences, which is generally more than adequate to uniquely tag each individual DNA molecule if the starting material is 10,000 GEs. Thus, at a given molar amount of synthesized primers, one could generate an almost infinite number of UIDs based on the length of the variable nucleotides. Using this approach, after PCR amplification, true mutations at low allele fraction should theoretically be present at nearly 100% of NGS reads within a barcode “family”; in addition, presuming there is enough starting DNA template of more than one mutant molecule, multiple barcode families should also be present that harbor the same mutation. Statistical algorithms via bioinformatics analyses dictate the number of mutant molecules in a given family, as well as the need for multiple families with the mutation, in order for one to call a true mutation with confidence (84). On the other hand, PCR and NGS artifacts would be present only in a single barcode family and likely with only a few NGS reads, and would therefore be discarded as artifacts. The LOD using these approaches can be as low as one mutant molecule per millions of wild-type DNA molecules, and indeed, studies have demonstrated that barcoded NGS approaches can even measure error rates of high-fidelity DNA polymerases estimated to be 1 error in 4.4 × 10^7^ nucleotides (84). The utilization and evolution of barcoding of DNA molecules have revolutionized the ability of NGS to detect rare mutations, and subsequently its use for clinical applications.

## Clinical utility

When discussing the current clinical utility of ctDNA for cancer care, one can envision two broad categories: qualitative and quantitative assessment. These are not mutually exclusive, but in general, only qualitative aspects of ctDNA have truly been utilized for clinical decision making thus far. If certain mutations are found in ctDNA of a patient with cancer, this opens the possibility of a targeted therapy — an on-label FDA-approved therapy, off-label use, and/or eligibility for a clinical trial. A recent example is mutant *PIK3CA* found in either tissue or ctDNA in patients with hormone receptor–positive, HER2-negative metastatic breast cancer, enabling the use of the PI3Kα inhibitor alpelisib (88), but there are many other examples beyond the scope of this Review. Qualitative aspects of ctDNA are also used to identify resistance mutations that serve as negative predictors of response to certain therapies. Examples include *KRAS* mutations, which predict for resistance to EGFR antibody–based therapies (89), as well as *ESR1* mutations in hormone receptor–positive breast cancers that predict for resistance to certain endocrine therapies (78, 90, 91). Although current commercial ctDNA assays are less sensitive than their tissue counterparts, this is largely due to pragmatic issues, i.e., the time and expense that would be needed for the level of redundant NGS, and the amount of blood/plasma and the extensive bioinformatics analyses required, for ctDNA assessment to approach the level of sensitivity of tumor tissue NGS. However, with newer technologies and with the cost of NGS continually decreasing (92), it is tempting to speculate that ctDNA may soon achieve a sensitivity of mutation detection equal if not superior to that of its tumor tissue counterpart.

As more genes and regions of the genome are sequenced using NGS, other qualitative/semiquantitative information becomes available that may also inform current clinical decision making. Specifically, so-called tumor mutation burden (TMB) and high microsatellite instability (MSI-H) are two tissue-agnostic FDA-approved indications for immunotherapy with the anti–PD-1 antibody pembrolizumab (93, 94). The reason TMB leads to a higher likelihood of response is thought to be the higher chance that any given mutation will create a neoantigen that can then be recognized by the immune system once anti–PD-1 therapy is initiated. This concept is similar to buying more lottery tickets; the more tickets purchased, the higher the odds of winning. Statistically, one would need to buy a critical number of lottery tickets to significantly impact the likelihood of winning. Similarly, a high TMB (defined as 10 mutations per megabase of DNA) implies a high number of mutations found within a cancer, which has been shown to be predictive of response to pembrolizumab owing to the increased likelihood of neoantigens that can be recognized by the immune system with anti–PD-1 therapy (94). Similarly, MSI-H tumors are generally cancers that arise from loss of mismatch repair gene function, leading to instability of microsatellite regions (repetitive DNA sequences) in the human genome that generally results in high TMB (93). Although MSI-H usually leads to a high TMB, high TMB can arise through other mechanisms. MSI-H is measured by NGS via evaluation of designated microsatellite regions and computation of whether these have enough changes in individual DNA strands to be classified as microsatellite stable, low, or high (95). Since the number of microsatellite regions queried is limited, ctDNA can be and is used by most commercial vendors to assess MSI-H. Because accurate assessment of TMB requires a large number of DNA base pairs to be sequenced, assessing TMB through NGS of ctDNA was historically challenging owing to the limitations mentioned above. However, with the increasing numbers of genes being sequenced using ctDNA, many commercial tests have recently incorporated this information into their liquid biopsy assays and thus can now be used to assess TMB and the likelihood of response to pembrolizumab and perhaps other immunotherapies (96).

Where ctDNA is still lacking is in the ability to use its quantitative aspects to guide clinical decisions. As mentioned previously, there have been and continue to be large numbers of studies using post hoc or planned analysis to evaluate the prognostic and predictive capability of ctDNA, in which plasma samples are analyzed at various time points retrospectively. However, despite these studies and their clinical validation, clinic utility is still lacking. Here, we are using Henry and Hayes’s definition of clinical utility (97), i.e., high-level evidence that quantitative detection of ctDNA can actually lead to changes and interventions that will positively affect outcomes for patients with cancer. Although such studies are ongoing and are the basis of many current clinical trials (98), these studies require long-term follow-up of outcomes akin to other screening/biomarker studies to definitively prove that disease-free, progression-free, and/or overall survival is meaningfully affected by the detection of ctDNA in undiagnosed people and/or patients with cancer. As an example, although quantitative assessment of circulating tumor cells (CTCs) has been clinically validated as a prognostic marker for metastatic breast cancer, its clinical utility has yet to be proven, and indeed, one study evaluating changing chemotherapy treatments based on a high number of CTCs did not show any benefit, i.e., showed lack of clinical utility (99). Further, any test that has been clinically validated as truly detecting cancer using ctDNA is ultimately not helpful for managing the disease until clinical utility has been proven — i.e., does acting on the results of the test improve outcomes in a clinically meaningful way?

Demonstrating clinical utility for ctDNA is especially challenging in studies evaluating ctDNA use as a primary cancer screening test, as well as for MRD in the adjuvant setting. As mentioned, a number of validation studies have been published or are ongoing, but to demonstrate that acting on these ctDNA results leads to improved outcomes such as the curing of more disease and/or improvement of overall survival will require intervention studies with long-term follow-up. Key to developing ctDNA assays for primary cancer screening and, in some instances, follow-up detection in the curative setting for MRD is knowledge that the ctDNA detected is coming from a given cancer type. For example, if one were to detect a *TP53* mutation in an asymptomatic person after cfDNA analysis, it would be unclear from which cell type this mutation arose, given that *TP53* mutations are found in a variety of human cancers. More perplexing, some such mutations, including *TP53*, may arise from clonal hematopoiesis (CH) (100). CH is relatively easy to detect using cfDNA but is often an incidental finding, as it has been shown that many asymptomatic individuals develop CH as a result of aging (101). Although CH may be the precursor to preleukemic syndromes, many individuals with CH will not experience any hematologic disease, and in fact, CH seems to have more correlation with cardiovascular risk than with hematologic cancer (102). In addition to the above concerns, the current sensitivity of ctDNA NGS assays can detect cancer cells prior to detection by radiographic scans; therefore, it is unclear what if anything should be done upon discovery of a ctDNA mutation in an asymptomatic person. To begin to address this vexing issue, recently investigators have explored the use of epigenetic DNA modifications and physical DNA fragmentation patterns to identify not only ctDNA, but which tissue of origin the ctDNA was derived from (103, 104). Bisulfite treatment followed by NGS is typically how one can distinguish methylated from unmethylated DNA (105), but newer approaches using immunoprecipitation have also been published and show high sensitivity and specificity for a given tumor type (106). These promising approaches set the stage for future clinical utility studies that may realize the full potential of ctDNA and liquid biopsies.

## Challenges and future directions

Despite the bright future and strong momentum of ctDNA as a tool to guide cancer prevention and therapy, many challenges remain. Beyond the need to prove clinical utility, decreasing turnaround time, decreasing costs, and improving sensitivity and specificity for MRD and primary cancer screening remain as technical, but not insurmountable, obstacles. As with most technologies, increased speed of NGS along with decreased costs continues to be the norm, such that one can easily envision that these issues will soon be resolved (92). However, the use of ctDNA for MRD, and as a primary cancer screening test, is more difficult to address. As mentioned, there are limited clinical validation studies attesting to the positive predictive value of ctDNA for these use indications. Detection of ctDNA generally is a harbinger of MRD as a primary screen and/or after curative intent therapies for patients with cancer. Again, further long-term studies are needed to prove that such detection is actionable and can result in clinically meaningful interventions that provide robust evidence of utility. On the other hand, there has been little or no validation regarding the negative predictive value of these tests, which implies that absence of ctDNA does not necessarily equate with lack of cancer. Thus, MRD may still exist in this scenario, and in the case of patients with cancer treated with curative intent, absence of ctDNA cannot currently be equated with cure. There are many technical reasons for this, and much of it depends on the nature of current assays designed to detect MRD. As an example, many ctDNA assays used to “track” MRD use a bespoke approach, meaning that tumor tissue from the patient is subjected to NGS and individual mutations are used as markers to follow response to therapies and/or MRD (62). In contrast, a generalized ctDNA assay using common mutated genes, and/or methylated versus unmethylated DNA that has been validated across various cancer types, may serve as a tissue-agnostic and independent approach for using blood to track and monitor ctDNA without the need for NGS of tumor tissue (59).

Both bespoke and generalized approaches have advantages and disadvantages and are not necessarily mutually exclusive. Although the bespoke assay lends further confidence and presumably sensitivity and specificity of individual mutations to be used as markers of MRD, this approach can be hampered by inadequacy (in both quantity and quality) of tissue samples as well as by the time needed to obtain tumor tissue, perform NGS to identify markers, and then develop individual markers for each patient. In certain clinical situations, this may not be practical or possible. On the other hand, although non-bespoke assays may forego the use of tissue NGS, a negative result may be indeterminant, since the mutated/methylated genes being queried may not be present in the genetic makeup of a given patient’s cancer.

**Figure 3:**
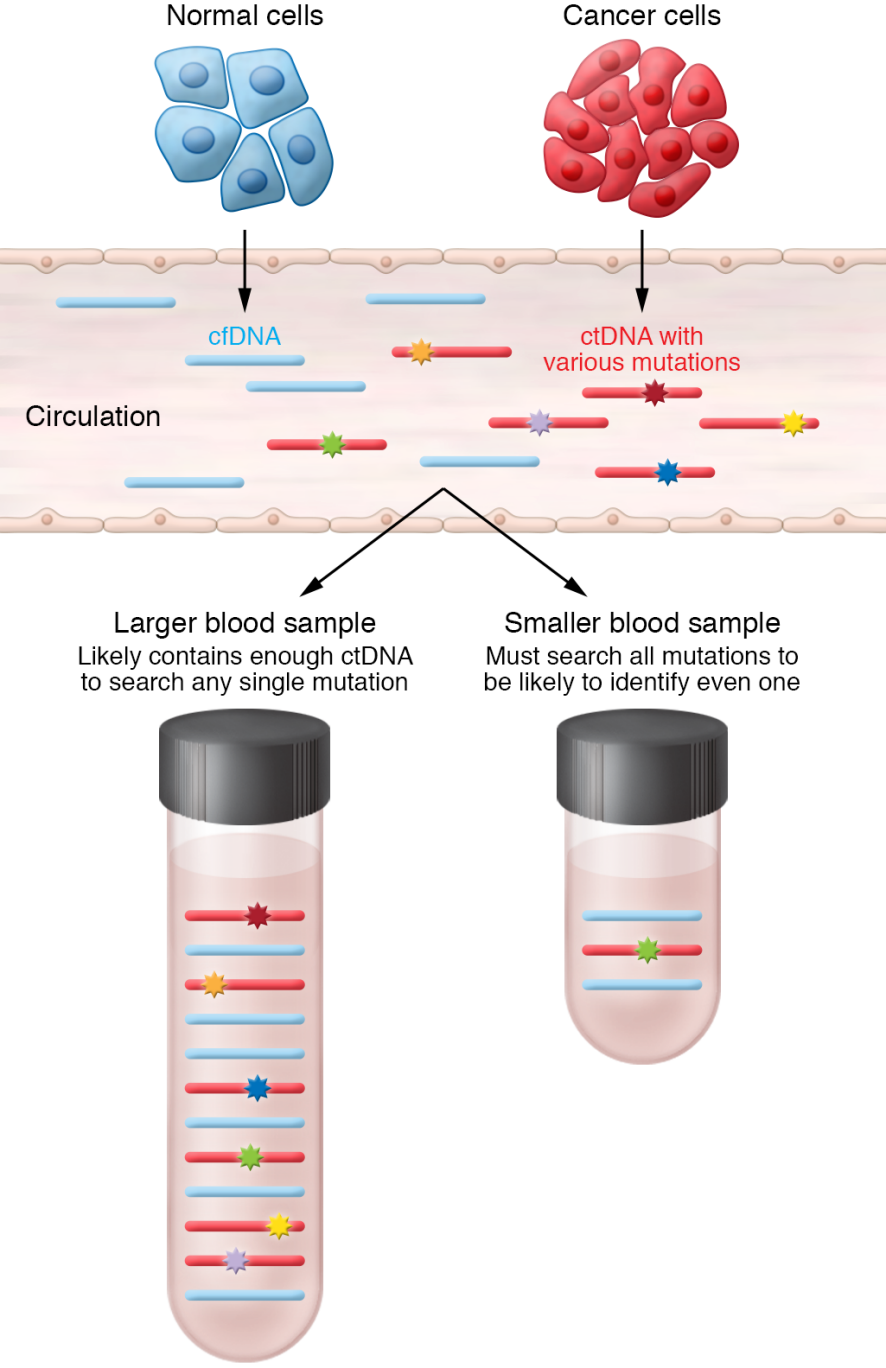
Rationale for increasing genome equivalents versus increasing the number of tracking mutations to identify ctDNA. In this example, the tumor/cancer cells have six distinct mutations (colored stars) that are being shed into the circulation along with normal DNA from normal cells (in blue). If an assay only queries for a single mutation, then a large amount of DNA is required to ensure a high likelihood that the mutation will be in the sample (bottom left). On the other hand, if plasma DNA is limited, then there may be only a single mutation in the sample (green star), and therefore querying for all DNA mutations is needed such that there is high likelihood that any mutation will be identified (bottom right).

Further obfuscation comes from the fact that a given plasma sample may not have enough GEs to decisively show that a negative result is truly free of ctDNA. There has been increasing interest in overcoming the limited number of GEs in a plasma sample by increasing the number of mutations or amount of methylated DNA for tracking of MRD (62). The concept here is that “more shots on goal” may allow for any rare ctDNA molecule to be identified because, for a given sample of plasma DNA, there may be only a few ctDNA molecules that may or may not contain a specific marker that is being tracked (Figure 3). Thus, rather than following one or two mutations, which would require tens of thousands of GEs, if enough mutations/methylated DNA markers are queried, the odds of detecting ctDNA may greatly increase even with limited amounts of plasma DNA. Although this approach was shown, in a limited manner, to have a very high positive predictive value (107), negative predictive value data are still lacking and may require a dramatic increase in the number of tracked markers to truly determine whether a negative result on a liquid biopsy test can allow reasonable confidence in identifying patients who are cured of their disease.

## Conclusions

In summary, liquid biopsies with ctDNA are a relatively new approach to help guide clinical decision making for the prevention, diagnosis, and treatment of human cancers. Applications of ctDNA as an indicator of disease status and mutational landscape are expanding, enabling oncologists to make more informed decisions with precision. Although liquid biopsies have become standard of care in select circumstances, there are still challenges and areas in which to grow its clinical utility for optimizing cancer care. Excitingly, one can envision ctDNA’s future role as a cancer screening test; as a companion diagnostic for obtaining targeted therapies and immunotherapies; for following response to therapies; and as a marker of MRD to determine which patients are potentially cured and which may benefit from additional therapies. Yet all of these applications and more can be easily obtained from a patient’s blood sample. Truly the future is bright for precision oncology with liquid biopsies at the forefront of this revolutionary way to make health care personal.

## Version 1 06/15/2022

Electronic publication
